# *PIK3CA *mutation impact on survival in breast cancer patients and in ERα, PR and ERBB2-based subgroups

**DOI:** 10.1186/bcr3113

**Published:** 2012-02-13

**Authors:** Magdalena Cizkova, Aurélie Susini, Sophie Vacher, Géraldine Cizeron-Clairac, Catherine Andrieu, Keltouma Driouch, Emmanuelle Fourme, Rosette Lidereau, Ivan Bièche

**Affiliations:** 1 Laboratoire d'Oncogénétique, Institut Curie, Hôpital René Huguenin, 35 Rue Dailly, Saint-Cloud, F-92210, France; 2 Laboratory of Experimental Medicine, Institute of Molecular and Translational Medicine, Faculty of Medicine and Dentistry, Palacky University and University Hospital Olomouc, Puskinova 6, Olomouc, 77520, Czech Republic; 3 Département d'Epidemiologie Clinique, Institut Curie, Hôpital René Huguenin, 35 Rue Dailly, Saint-Cloud, F-92210, France

## Abstract

**Introduction:**

*PIK3CA *is the oncogene showing the highest frequency of gain-of-function mutations in breast cancer, but the prognostic value of *PIK3CA *mutation status is controversial.

**Methods:**

We investigated the prognostic significance of *PIK3CA *mutation status in a series of 452 patients with unilateral invasive primary breast cancer and known long-term outcome (median follow-up 10 years).

**Results:**

*PIK3CA *mutations were identified in 151 tumors (33.4%). The frequency of *PIK3CA *mutations differed markedly according to hormone receptor (estrogen receptor alpha [ERα] and progesterone receptor [PR]) and ERBB2 status, ranging from 12.5% in the triple-negative subgroup (ER-/PR-/ERBB2-) to 41.1% in the HR+/ERBB2- subgroup. *PIK3CA *mutation was associated with significantly longer metastasis-free survival in the overall population (P = 0.0056), and especially in the PR-positive and ERBB2-positive subgroups. In Cox multivariate regression analysis, the prognostic significance of *PIK3CA *mutation status persisted only in the ERBB2-positive subgroup.

**Conclusions:**

This study confirms the high prevalence of *PIK3CA *mutations in breast cancer. *PIK3CA *mutation is an emerging tumor marker which might become used in treatment-choosing process. The independent prognostic value of *PIK3CA *mutation status in ERBB2-positive breast cancer patients should be now confirmed in larger series of patients included in randomized prospective ERBB2-based clinical trials.

## Introduction

Dysregulation of tyrosine kinase receptor (TKR)-phosphatidylinositol 3-kinase (PI3K) signaling pathways is frequent in human cancers. Among the most important molecular events downstream of TKR activation is PI3K activation, which catalyzes the phosphorylation of inositol lipids to phosphatidylinositol-3,4,5-trisphosphate. Phosphatidylinositol-3,4,5-trisphosphate activates the serine/threonine kinase AKT, which in turn regulates several signaling pathways controlling cell survival, apoptosis, proliferation, motility, and adhesion [1]. PI3K is a heterodimeric enzyme composed of a p110α catalytic subunit encoded by the *PIK3CA *gene and a p85 regulatory subunit encoded by the *PIK3R1 *gene [2].

Recently, gain-of-function mutations in *PIK3CA *have been found in several cancers, including breast cancer [1,3,4]. *PIK3CA *is frequently mutated at 'hotspots' in exons 9 and 20, corresponding to the helical (E542K and E545K) and kinase (H1047R) domains, respectively. P110α carrying a hotspot mutation shows oncogenic activity: it can transform primary fibroblasts in culture, induce anchorage-independent growth, and cause tumors in animals [5,6].

After the *TP53 *suppressor gene, the *PIK3CA *oncogene is the most frequently mutated gene in human breast cancers; mutations are observed in 20% to 40% of cases [7,8]. Mutation is an early event in breast cancer and is more likely to play a role in tumor initiation than in invasive progression [9]. It is noteworthy that activating somatic mutations of other oncogenes (*EGFR, KRAS, HRAS, NRAF, BRAF, AKT1*, and so on) involved in molecular events downstream of TKR activation and frequently observed in other cancers are rare in breast cancer. Several studies of breast cancer suggest that *PIK3CA *mutations are more frequent in estrogen receptor-alpha- positive (ERα^+^) breast tumors (30% to 40%) than in receptor-alpha-negative (ERα^-^) breast tumors (10% to 20%) [3,7,10,11].

The prognostic value of *PIK3CA *mutation status in breast cancer is controversial. Li and colleagues [12] suggested that mutations in any part of the gene may be related to poor clinical outcome. On the contrary, Maruyama and colleagues [13], Pérez-Tenorio and colleagues [14], and Kalinsky and colleagues [11] suggested that *PIK3CA *mutations were significantly and independently associated with better recurrence-free survival. In particular, Kalinsky and colleagues [11] studied a series of 590 patients with breast cancer with a median follow-up of 12.8 years and found 32.5% of *PIK3CA *mutations. *PIK3CA*-mutated status was associated with markers of good prognosis and with significant improvement in overall (*P *= 0.03) and breast cancer-specific (*P *= 0.004) survival [11]. A study focused specifically on recurrent and metastatic breast cancer found a significant association of *PIK3CA *mutations and longer relapse-free survival [15]. Barbareschi and colleagues [16] reported that only *PIK3CA *exon 9 mutations were independently associated with early recurrence and death but that exon 20 mutations were associated with favorable outcome. Several teams have found no significant effect of *PIK3CA *mutations on patient outcome [7,8,17,18]. It is, however, noteworthy that Loi and colleagues [18] identified an expression signature derived from exon 20 *PIK3CA*-mutated tumors. This signature predicted better outcome in ER^+ ^breast cancer. In particular, the clinical consequences of *PIK3CA *mutations might vary according to the status of well-known molecular markers in breast cancer, namely *ERα*, progesterone receptor (*PR*), and *ERBB2*. Here, we examined the prognostic value of *PIK3CA *mutation status in a series of 452 patients with unilateral invasive primary breast cancer and known long-term outcome, taking *ERα, PR*, and *ERBB2 *status into account.

## Materials and methods

### Patients and samples

We analyzed samples of 452 primary unilateral invasive primary breast tumors excised from women at the Institut Curie/Hôpital René Huguenin (Saint-Cloud, France) from 1978 to 2008. All patients who entered our institution before 2007 were informed that their tumor samples might be used for scientific purposes and had the opportunity to decline. Since 2007, patients entering our institution have given their approval also by signed informed consent. This study was approved by the local ethics committee (Breast Group of René Huguenin Hospital). The samples were examined histologically and were considered suitable for this study if the proportion of tumor cells exceeded 70% with sufficient cellularity as was proven by evaluation of tumor samples stained by hematoxylin and eosin. Immediately after surgery, the tumor samples were placed in liquid nitrogen until RNA extraction.

The patients (mean age of 61.6 years and range of 31 to 91) met the following criteria: primary unilateral non-metastatic breast carcinoma, with full clinical, histological and biological data; no radiotherapy or chemotherapy before surgery; and full follow-up at Institut Curie/Hôpital René Huguenin.

One hundred sixty patients (35.4%) had breast-conserving surgery plus locoregional radiotherapy, and 292 patients (64.6%) had modified radical mastectomy. Clinical examinations were performed every 3 or 6 months during the first 5 years, according to the prognostic risk of the patients, and then yearly. Mammograms were done annually. Three hundred sixty-six patients received adjuvant therapy, consisting of chemotherapy alone in 94 cases, hormone therapy alone in 177 cases, and both treatments in 95 cases. None of the ERBB2^+ ^patients was treated with anti-ERBB2 therapy. The histological type and number of positive axillary nodes were established at the time of surgery. The malignancy of infiltrating carcinomas was scored with the Scarff-Bloom-Richardson histoprognostic system'.

ER and PR status was determined at the protein level by using biochemical methods (dextran-coated charcoal method or enzymatic immunoassay) until 1999 and later by using immunohistochemistry. Cutoff for ER and PR positivity was set at 15 fm/mg (dextran-coated charcoal or enzyme immunoassay) and at 10% immunostained cells (immunohistochemistry). A tumor was considered ERBB2^+ ^by immunohistochemistry if it scored 3 or more with uniform intense membrane staining of greater than 30% of invasive tumor cells. Tumors scoring 2 or more were considered to be equivocal for ERBB2 protein expression and were tested by fluorescence *in situ *hybridization for ERBB2 gene amplification. In all cases, the *ERα, PR*, and *ERBB2 *status was confirmed by real-time quantitative reverse transcriptase-polymerase chain reaction (RT-PCR) with cutoff levels based on previous studies comparing results of the mentioned methods [19-22]. On the basis of hormone receptor (HR) (*ERα *and *PR*) and *ERBB2 *status, we subdivided the 452 patients into four subgroups: HR^+ ^(ER^+ ^or PR^+ ^or both)/ERBB2^+ ^(*n *= 53), HR^+ ^(ER^+ ^or PR^+ ^or both)/ERBB2^- ^(*n *= 287), HR^- ^(ER^- ^and PR^-^)/ERBB2^+ ^(*n *= 48), and HR^- ^(ER^- ^and PR^-^)/ERBB2^- ^(*n *= 64). Standard prognostic factors are reported in Table S1 of Additional file 1. The median follow-up was 10.0 years (range of 13 months to 28.9 years). One hundred seventy patients developed metastases.

### RNA extraction

Total RNA was extracted from breast tumor samples by using the acid-phenol guanidium method. RNA quantity was assessed by using a NanoDrop Spectrophotometer ND-1000 with its corresponding software (Thermo Fisher Scientific Inc., Wilmington, DE, USA). RNA quality was determined by electrophoresis through agarose gel and staining with ethidium bromide. The 18S and 28S RNA bands were visualized under ultraviolet light. DNA contamination was quantified by using a couple of primers located in an intron of gene coding for albumin (ALB) (Gene ID: 213). Samples were further used only when the cycle threshold (Ct) obtained by using these ALB intron primers was greater than 40.

### *PIK3CA *mutation screening

*PIK3CA *mutations were detected by screening cDNA fragments obtained by RT-PCR amplification of exons 9 and 20 and their flanking exons. Details of the primers and PCR conditions are available on request. The amplified products were sequenced with a BigDye Terminator kit on an ABI Prism 3130 automatic DNA sequencer (Applied Biosystems, Courtabæuf, France) with detection sensitivity of 5% mutated cells, and the sequences were compared with the corresponding cDNA reference sequence (NM_006218). All of the detected *PIK3CA *mutations were confirmed in the second independent run of sample testing.

### Statistical analysis

Relationships between *PIK3CA *mutation status and clinical, histological, and biological parameters were estimated with the chi-squared test. Differences between the mutated and non-mutated populations were judged significant at confidence levels of greater than 95% (*P *< 0.05). Metastasis-free survival (MFS) was determined as the interval between diagnosis and detection of the first metastasis. Survival distributions were estimated with the Kaplan-Meier method [23], and the significance of differences between survival rates was ascertained with the log-rank test [24]. The Cox proportional hazards regression model [25] was used to assess prognostic significance.

## Results and Discussion

*PIK3CA *mutations were identified in 151 (33.4%) of 452 primary breast tumors, in keeping with the results of the largest previous studies, showing mutation rates of 25% to 40% [7,8,11,14,16,18,26-30]. Sixty-four tumors bore *PIK3CA *mutations located in exon 9, 86 tumors bore mutations in exon 20, and one tumor bore mutations in both exons 9 and 20 (Table 1). Exon 20 was thus the most frequently mutated *PIK3CA *exon, in keeping with most other studies [7,8,11,14,26,28-30]. Among the 151 tumors with *PIK3CA *mutations, three bore double mutations: two in exon 20 (D1029H and H1047R, H1047R and A1066V) and one in exons 9 and 20 (E542K and M1043V). Rare double *PIK3CA *mutations have been reported elsewhere [7,8,30]. We also observed two c.3203dupA frameshift mutations that would change the last C-terminal amino acid (N1068K) of the PIK3CA protein and add another three amino acids. N1068K represents 50% of all *PIK3CA *mutations in hepatocellular carcinoma [28] but its possible role in tumor initiation or progression is unknown.

**Table 1:** *PIK3CA *mutation profiles

Exon	Nucleotide	Codon	Number of mutations
9	c.1634A > C	Glu545Ala	2
9	c.1636C > A	Gln546Lys	2
9	c.1624 G > A	Glu542Lys	20
9	c.1634A > G	Glu545Gly	1
9	c.1633 G > A	Glu545Lys	32
9	c.1633 G > C	Glu545Gln	1
9	c.1490A > G	Asn497Ser	1
9	c.1636C > A	Gln546Lys	2
9	c.1637A > C	Gln546Pro	1
9	c.1637A > G	Gln546Arg	2
20	c.3203dupA	Asn1068Lys	2
20	c.3140A > T	His1047Leu	8
20	c.3140A > G	His1047Arg	70
20	c.3132T > A	Asn1044Lys	1
20	c.3145 G > C	Gly1049Arg	2
20	c.3155C > A	Thr1052Lys	1
20	c.[3085 > C(+)3140A > T]	p.[Asp1029His(+)His1047Leu]	1
20	c.[3140A > T(+)3197C > T]	p.[His1047Leu(+)Ala1066Val]	1
9+20	c.[1624 G < A(+)3127A > G]	p.[Glu542Lys(+)Met1043Val]	1
			Total = 151

*PIK3CA*, phosphatidylinositol 3-kinase, catalytic, alpha polypeptide gene.

Table 2 shows links between *PIK3CA *mutation status and standard clinical, pathological, and biological characteristics of breast cancer. *PIK3CA *mutations were significantly associated (chi-squared test) with low histopathological grade, small macroscopic tumor size, and ERα^+^, PR^+^, and ERBB2^- ^tumors. For example, *PIK3CA *mutations were observed in 52.7% (29 out of 55) of histopathological grade I tumors, 36.8% (84 out of 228) of grade II tumors, and 23.3% (37 out of 159) of grade III tumors. These relationships have also been found in most previous studies [3,7,10,11]. For example, Kalinsky and colleagues [11], like us, found that *PIK3CA *mutations were associated with low histopathological grade and ERα^+^, PR^+^, and ERBB2^- ^tumors. However, it is noteworthy that, in several studies, no significant association between *PIK3CA *mutations and important clinical or pathological features was found [30]. A high frequency of *PIK3CA *mutations has also been found in lobular carcinoma [16,31]. In agreement with other authors [27,30], we observed a similar frequency of *PIK3CA *mutations in lobular carcinomas (34.5%, 10 out of 29) and ductal carcinomas (33.2%, 129 out of 388) of the breast (Table 2).

**Table 2:** Relationship between *PIK3CA *mutation status and standard clinical, pathological, and biological features of breast cancer

		Number of patients (percentage)		
	Total population number (percentage)	*PIK3CA *wild-type	*PIK3CA*-mutated	*P *value^a^
Total	452 (100.0)	301 (66.6)	151 (33.4)	
Age, years				
≤ 50	96 (21.2)	66 (21.9)	30 (19.9)	NS
> 50	356 (78.8)	235 (78.1)	121 (81.1)	
SBR histological grade^b, c^				
I	55 (12.4)	26 (8.9)	29 (19.3)	0.00021
II	228 (51.6)	144 (49.3)	84 (56.0)	
III	159 (36.0)	122 (41.8)	37 (24.7)	
Lymph node status^d^				
0	115 (25.5)	78 (26.0)	37 (24.5)	
1-3	237 (52.5)	157 (52.3)	80 (53.0)	
> 3	99 (22.0)	65 (21.7)	34 (22.5)	NS
Macroscopic tumor size^e^				
≤ 25 mm	217 (48.8)	135 (45.2)	82 (56.2)	0.029
> 25 mm	228 (51.2)	164 (54.8)	64 (43.8)	
ERα status				
Negative	117 (25.9)	97 (32.2)	20 (13.2)	0.000014
Positive	335 (74.1)	204 (67.8)	131 (86.8)	
PR status				
Negative	194 (42.9)	150 (49.8)	44 (29.1)	0.000028
Positive	258 (57.1)	151 (50.2)	107 (70.9)	
ERBB2 status				
Negative	351 (77.7)	225 (74.8)	126 (83.4)	0.036
Positive	101 (22.3)	76 (25.2)	25 (16.6)	
Histology				
Ductal	388 (85.8)	259 (86.0)	129 (85.5)	NS
Lobular	29 (6.4)	19 (6.3)	10 (6.6)	
Others	35 (7.8)	23 (7.7)	12 (7.9)	

^a^Chi-squared test. ^b^Scarff-Bloom-Richardson (SBR) classification. ^c^Information available for 442 patients. ^d^Information available for 451 patients. ^e^Information available for 445 patients. ERα, estrogen receptor-alpha; NS, not significant; *PIK3CA*, phosphatidylinositol 3-kinase, catalytic, alpha polypeptide gene; PR, progesterone receptor.

Functional genomic studies have recently shown that breast cancer is a highly heterogeneous disease. Several tumor subtypes, such as basal-like, *ERBB2*^+^, and HR^+ ^(luminal A and luminal B), can be distinguished on the basis of their gene expression profiles, pointing to the involvement of different oncogenetic pathways. In keeping with this possibility, we observed a marked difference in the *PIK3CA *mutation frequency across four major tumor subgroups: HR^+^/ERBB2^+ ^(28.3%, 15 out of 53), HR^+^/ERBB2^- ^(41.1%, 118 out of 287), HR^-^/ERBB2^+ ^(20.8%, 10 out of 48), and HR^-^/ERBB2^- ^(12.5%, 8 out of 64) (*P *= 0.00009). Being found in 41.1% of cases, *PIK3CA *mutations might thus be characteristic of the luminal subtype (HR^+^/ERBB2^-^). We also observed a low frequency (12.5%) of *PIK3CA *mutations in triple-negative tumors (ER^-^/PR^-^/ERBB2^-^), a subgroup reported to overlap with the basal-like subtype of breast cancer. Stemke-Hale and colleagues [8] also observed a marked difference in *PIK3CA *mutation frequency across breast tumor subtypes, and *PIK3CA *mutations were more common in HR^+ ^tumors (39%) and ERBB2^+ ^tumors (25%) than in basal-like tumors (13%).

**Figure 1:**
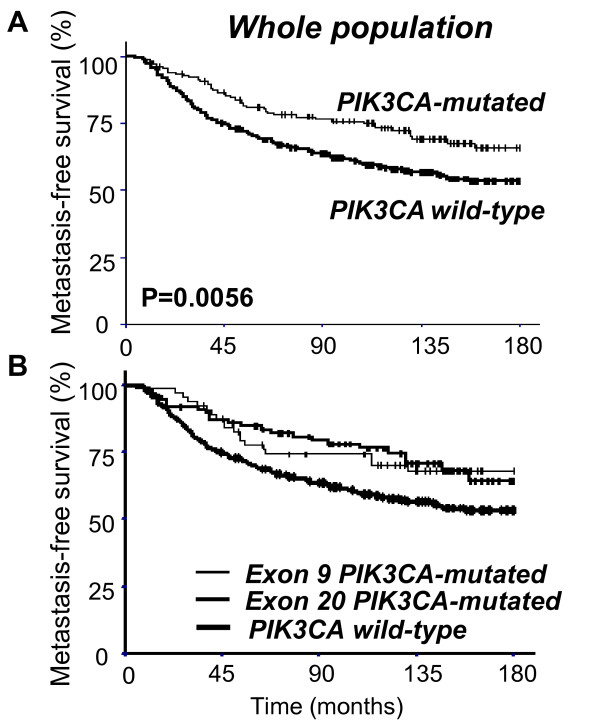
**Whole population survival curves**. **(a) **Metastasis-free survival curves of patients with *PIK3CA *wild-type and -mutated tumors. **(b) **Metastasis-free survival curves of patients with exon 9 *PIK3CA*-mutated tumors, exon 20 *PIK3CA*-mutated tumors, and *PIK3CA *wild-type tumors. Comparison of these curves did not show any statistically significant difference. *PIK3CA*, phosphatidylinositol 3-kinase, catalytic, alpha polypeptide gene.

In the overall population of 452 patients, *PIK3CA *mutation was associated with more favorable MFS (*P *= 0.0056) (Table 3 and Figure 1a). The outcome of the 151 patients with *PIK3CA *mutations was thus significantly better than that of the 301 wild-type patients, as was demonstrated by 5-year and 15-year survival rates in these two groups (5-year MFS of 81.0% versus 69.6% and 15-year MFS of 65.8% versus 53.4%). Differences in treatment are unlikely to account for this difference, as *PIK3CA *mutations were as frequent in patients who received postoperative adjuvant chemotherapy or hormone therapy or both (126 out of 366, 34.4%) as in those who received neither treatment (25 out of 86, 29.1%).

**Table 3:** *PIK3CA *mutation status according to hormone receptor and ERBB2 status and relation to metastasis-free survival

	Number of patients	5-year MFS	HR (95% CI)	*P *value^a^
Total population	452			
Wild-type	301	69.6%	1	0.0056
Mutated	151	81.0%	0.62 (0.44-0.87)	
ERα^+^	335			
Wild-type	204	75.6%	1	NS
Mutated	131	81.9%	0.71 (0.49-1.04)	
ERα^-^	117			
Wild-type	97	56.9%	1	NS
Mutated	20	75.0%	0.46 (0.18-1.15)	
PR^+^	258			
Wild-type	151	77.8%	1	0.0064
Mutated	107	86.6%	0.52 (0.33-0.83)	
PR^-^	194			
Wild-type	150	61.3%	1	NS
Mutated	44	67.6%	0.91 (0.55-1.50)	
ERBB2^+^	101			
Wild-type	76	59.9%	1	0.014
Mutated	25	88.0%	0.31 (0.12-0.79)	
ERBB2^-^	351			
Wild-type	225	72.9%	1	NS
Mutated	126	79.7%	0.75 (0.51-1.08)	

^a^Univariate Cox analysis. CI, confidence interval; ERα, estrogen receptor-alpha; HR, hazard ratio; MFS, metastasis-free survival; NS, not significant; PR, progesterone receptor.

These data confirm the results of smaller series of breast tumors, in which *PIK3CA *mutations were significantly associated with more favorable MFS [13,14]. However, unlike Barbareschi and colleagues [16], who found that mutations in the helical (exon 9) and kinase (exon 20) domains of the *PIK3CA *gene had different prognostic values, we found that MFS was similar in patients with mutations in one exon or the other when we compared these two subgroups together and with the wild-type subgroup (Figure 1b).

**Figure 2:**
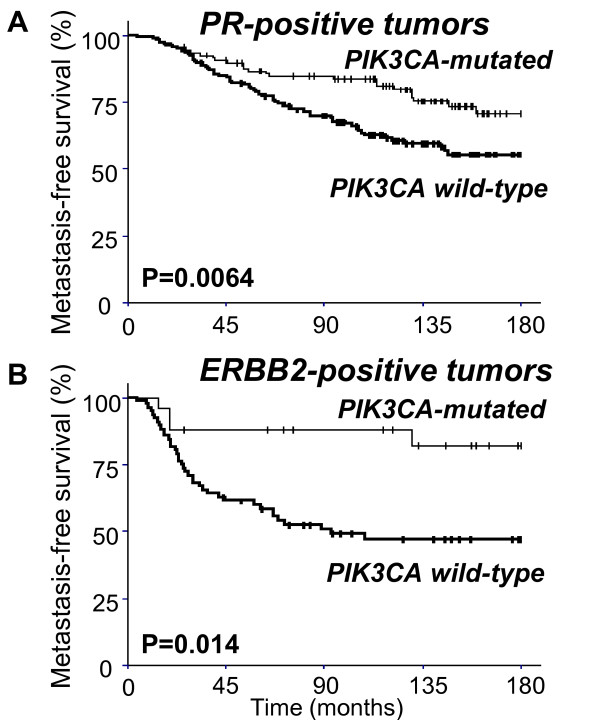
**Subgroup analysis survival curves**. **(a) **Metastasis-free survival curves of progesterone receptor-positive (PR^+^) patients with *PIK3CA *wild-type and -mutated tumors. **(b) **Metastasis-free survival curves of ERBB2^+ ^patients with *PIK3CA *wild-type and -mutated tumors. *PIK3CA*, phosphatidylinositol 3-kinase, catalytic, alpha polypeptide gene.

More interestingly, *PIK3CA *mutation was associated with markedly better MFS in the patients with PR^+ ^tumors (*P *= 0.0064) than in those with PR^- ^tumors (*P *= 0.71) (Table 3 and Figure 2a) and also in patients with ERBB2^+ ^tumors (*P *= 0.014) than in those with ERBB2^- ^tumors (*P *= 0.12) (Table 3 and Figure 2b). In contrast, *PIK3CA *mutation was associated only with a trend toward better MFS in patients with ERα^+ ^(*P *= 0.082) and ERα^- ^(*P *= 0.098) tumors (Table 3). Accordingly, Loi and colleagues [18] did not find statistically significant difference in survival between *PIK3CA *wild-type and *PIK3CA*-mutated tumors in the ER^+ ^population. However, it is noteworthy that these authors described a *PIK3CA *mutation-associated gene expression signature predicting favorable survival in ER^+ ^breast cancer [18].

Using a Cox proportional hazards model, we also assessed the MFS predictive value of the parameters that were significant in univariate analysis (that is, Scarff-Bloom-Richardson histological grade, lymph node status, macroscopic tumor size, and ERα, PR, and ERBB2 status (Table S1 of Additional file 1) and *PIK3CA *mutation status). The prognostic significance of *PIK3CA *mutation status persisted in the ERBB2^+ ^tumor subgroup (*P *= 0.023) (Table 4) but not in the total tumor population or in the PR^+ ^tumor subgroup. Since the patients were not treated with ERBB2-targeted treatment, these results address the outcome of ERBB2^+ ^tumors affected by surgery and chemotherapy but not targeted therapy like trastuzumab or lapatinib. The independent prognostic value of *PIK3CA *mutation status in patients with ERBB2^+ ^breast cancer should now be tested in a larger series of patients included in randomized prospective ERBB2-based clinical trials.

**Table 4:** Multivariate Cox analysis of metastasis-free survival in the total population and in subgroups of patients with breast cancer

Variables	Total population		ER^+ ^patients		ER^- ^patients		PR^+ ^patients		PR^- ^patients		ERBB2^+ ^patients		ERBB2^- ^patients	
	HR (95% CI)	*P *value^a^	HR (95% CI)	*P *value^a^	HR (95% CI)	*P *value^a^	HR (95% CI)	*P *value^a^	HR (95% CI)	*P *value^a^	HR (95% CI)	*P *value^a^	HR (95% CI)	*P *value^a^
SBR		0.038		0.019		NS		0.00077		NS		NS		0.0043
I	1		1		1		1		1		1		1	
II	1.34 (1.02-1.76)		1.45 (1.06-1.98)		1.02 (0.57-1.80)		1.88 (1.30-2.71)		0.93 (0.63-1.38)		0.89 (0.51-1.55)		1.60 (1.16-2.21)	
III	1.79 (1.03-3.11)		2.11 (1.13-3.92)		1.03 (0.33-3.26)		3.52 (1.69-7.32)		0.87 (0.40-1.91)		0.79 (0.26-2.39)		2.56 (1.34-4.90)	
pN		0.00014		0.00093		NS		0.0068		0.01		0.000049		NS
0	1		1		1		1		1		1		1	
1-3	1.58 (1.25-1.99)		1.60 (1.21-2.11)		1.45 (0.94-2.25)		1.61 (1.14-2.27)		1.51 (1.10-2.07)		2.61 (1.63-4.18)		1.26 (0.97-1.66)	
> 3	2.48 (1.56-3.96)		2.56 (1.47-4.47)		2.11 (0.88-5.05)		2.59 (1.30-5.18)		2.28 (1.21-4.29)		6.83 (2.67-17.44)		1.60 (0.93-2.74)	
pT		0.01		0.00041		NS		0.0023		NS		NS		0.0054
≤ 25 mm	1		1		1		1		1		1		1	
> 25 mm	1.53 (1.11-2.13)		2.05 (1.38-3.05)		0.80 (0.44-1.47)		2.09 (1.30-3.36)		1.17 (0.74-1.84)		1.06 (0.54-2.08)		1.72 (1.17-2.52)	
*ER*		NS		-		-		NS		NS		NS		NS
Negative	1		-		-		1		1		1		1	
Positive	1.04 (0.68-1.60)		-		-		2.09 (0.28-15.63)		0.82 (0.51-1.32)		0.78 (0.36-1.70)		1.23 (0.71-2.11)	
*PR*		NS		NS		NS		-				NS		NS
Negative	1		1		1		-		-		1		1	
Positive	0.77 (0.52-1.14)		0.81 (0.54-1.23)		0.31 (0.04-2.33)		-		-		0.56 (0.23-1.36)		0.79 (0.51-1.23)	
*ERBB2*		NS		NS		NS		NS		NS		-		-
Negative	1		1		1		1		1		-		-	
Positive	1.12 (0.77-1.62)		1.01 (0.60-1.70)		1.33 (0.74-2.38)		0.98 (0.50-1.93)		1.17 (0.74-1.85)		-		-	
*PIK3CA*		NS		NS		NS		NS		NS		0.023		NS
Wild-type	1		1		1		1		1		1		1	
Mutated	0.75 (0.53-1.07)		0.84 (0.57-1.23)		0.49 (0.19-1.26)		0.62 (0.38-1.01)		0.92 (0.55-1.54)		0.31 (0.12-0.79)		0.96 (0.65-1.44)	

^a^Multivariate Cox analysis. CI, confidence interval; ER, estrogen receptor; HR, hazard ratio; NS, not significant; *PIK3CA*, phosphatidylinositol 3-kinase, catalytic, alpha polypeptide gene; pN, number of positive axillary lymph nodes assessed by a pathologist at the time of surgery; PR, progesterone receptor; pT, size of the primery tumor assessed by a patologist at the time of surgery}; SBR, Scarff-Bloom-Richardson classification.

*PIK3CA *mutation is also an emerging tumor marker that, in the future, might be used in the process of choosing a treatment. Indeed, ERBB2 inhibitors (trastuzumab and lapatinib) are clinically active in women with ERBB2^+ ^breast cancer, but recent studies suggest that *PIK3CA*-mutated tumors could be resistant to these drugs [32,33]. There is also evidence showing that tumors with PI3K/AKT pathway activation including PTEN loss or *PIK3CA *mutation or both are less sensitive to trastuzumab treatment [17]. Interestingly, this resistance appears to be reversed by mammalian target of rapamycin (mTOR) or PI3K inhibitors [33]. A final validation of *PIK3CA *mutation as an independent predictor of the response to trastuzumab treatment in ERBB2^+ ^breast cancer needs a prospective randomized study. Our results also support the emerging role of *PIK3CA *mutation status in the management of future gene-based therapies (ERBB2, mTOR, or PI3K inhibitors used alone or in combination) for breast cancer, particularly in patients with tumors with activated PI3K/AKT pathway [34,35]. *ERBB2 *amplification and *PIK3CA *mutation were recently validated as biomarkers of sensitivity to single-agent PI3K inhibitor (GDC-0941) therapy in breast cancer models [35].

## Conclusions

This study of 452 breast tumors confirms the high prevalence (33.4%) of *PIK3CA *mutations. The frequency of *PIK3CA *mutations differed markedly according to *ERα, PR*, and *ERBB2 *status, from 12.5% in triple-negative tumors to 41.1% in the HR^+^/ERBB2^- ^subgroup. Subgroup analysis of patient survival identified *PIK3CA *mutation status as an independent prognostic value in patients with ERBB2^+ ^breast cancer. These findings should be confirmed in larger series of patients included in a randomized prospective ERBB2-based clinical trial. Then *PIK3CA *mutation status could serve as a new independent prognostic tool when selecting targeted therapies for patients with ERBB2^+ ^breast cancer.

## Abbreviations

ALB: albumin; ERα: estrogen receptor-alpha; HR: hormone receptor; MFS: metastasis-free survival; mTOR: mammalian target of rapamycin; PCR: polymerase chain reaction; PI3K: phosphatidylinositol 3-kinase; *PIK3CA*: phosphatidylinositol 3-kinase: catalytic: alpha polypeptide gene; PR: progesterone receptor; RT-PCR: reverse transcriptase-polymerase chain reaction; TKR: tyrosine kinase receptor.

## Competing interests

The authors declare that they have no competing interests.

## Authors' contributions

AS and SV helped to conceive the approach to mutational analysis, design the primers, and carry out the mutational analysis. CA helped to conceive the approach to mutational analysis, design the primers, and perform the DNA extraction. MC helped to carry out the mutational analysis and draft the manuscript. GC-C and EF performed the statistical analysis. IB and RL helped to draft the manuscript and conceive the study and participated in its design and coordination. KD helped to draft the manuscript. All authors read and approved the final manuscript.

## Supplementary Material

Additional file 1 **Table S1**. Characteristics of the 452 primary breast tumors, and relation to metastasis-free survival. A table showing metastasis free survival of the patients in relation to pathological data. Click here for file
