# Supplementary material for: PIK3CA mutation impact on survival in breast cancer patients and in ERα, PR and ERBB2-based subgroups
Source: Breast Cancer Res. 2012 Feb 13;14(1):R28. doi: 10.1186/bcr3113 (PMC3496146; doi:10.1186/bcr3113)
Supplement: Additional file 1 — Table S1. Characteristics of the 452 primary breast tumors, and relation to metastasis-free survival. A table showing metastasis free survival of the patients in relation to pathological data. [file bcr3113-S1.PDF]

Additional Table S1 Characteristics of the 452 primary breast tumors, and relation to metastasis-free survival.

	Number of patients	5-y MFS	*P*-value[a]
Total	452	73.4 %	-
Age			
≤50	96	83.3 %	NS
>50	356	80.5 %	
SBR histological grade[b,c]			
I	55	92.5 %	
II	228	75.3 %	**0.000064**[f]
III	159	64.3 %	
Lymph node status[d]			
0	115	79.0 %	
1-3	237	76.8 %	**0.00000026**[f]
>3	99	58.6 %	
Macroscopic tumor size[e]			
≤25mm	217	82.7 %	**0.000011**
>25mm	228	64.1 %	
ERα			
Negative	117	60.1 %	**0.0046**
Positive	335	78.1 %	
PR			
Negative	194	62.6 %	**0.0007**
positive	258	81.4 %	
ERBB2			
Negative	351	75.3 %	NS
Positive	101	66.8 %	
HR/ERBB2 subgroups			
HR+ ERBB2+	53	79.0 %	
HR + ERBB2-	287	78.3 %	**0.0049**[f]
HR - ERBB2+	48	53.4 %	
HR - ERBB2 -	64	62.0 %	
Histology			
Ductal	388	73.5 %	
Lobular	29	79.3 %	NS[f]
Others	35	68.3 %	

[a]Log-rank test. NS: not significant.

[b]Scarff Bloom Richardson classification.

[c]Information available for 442 patients.

[d]Information available for 451 patients.

[e]Information available for 445 patients.

[f]Global comparison of all subgroups of a category.
